# Chronic Kidney Disease—State of Either “Too Much” or “Too Little”

**DOI:** 10.3390/nu15071587

**Published:** 2023-03-24

**Authors:** Vincent Mathias Brandenburg, Turgay Saritas

**Affiliations:** 1 Department of Cardiology and Nephrology, Rhein-Maas Klinikum Würselen, D-52146 Wuerselen, Germany; 2 Division of Nephrology and Clinical Immunology, Department of Internal Medicine, University Hospital RWTH Aachen, D-52057 Aachen, Germany; 3 Institute of Experimental Medicine and Systems Biology, University Hospital RWTH Aachen, D-52057 Aachen, Germany

Chronic kidney disease (CKD) is a world-wide phenomenon with an increasing incidence and prevalence. In 2017, the global prevalence of CKD was 9.1%, which corresponds to roughly 700 million cases [1]. CKD is much more than one or more lab values diverging from the normal range. Compared with other risk factors, reduced glomerular filtration rate (GFR)—a key feature of CKD—is an independent risk factor for cardiovascular disease and overall mortality: A large population-based registry analysis clearly indicated the presence of an independent, graded association between a reduced estimated GFR and the risk of death, cardiovascular events, and hospitalization [2]. CKD was the 12th leading cause of death globally in 2017, representing an increase from 17th place in 1990. Predictions have suggested that CKD will become the fifth highest cause of death globally by 2040 [3]. Notably, these numbers are most likely underestimated due to missing systematic population-wide screening programs for CKD. Common diseases often related to a Western life style, such as the epidemic occurrence of arterial hypertension and diabetes mellitus, are among the driving forces behind CKD development and progression. Hence, it appears unlikely that the number of people with overt or hidden CKD will diminish in the upcoming decades.

The basic roles of the kidneys are maintaining body homeostasis by the regulation of water and electrolyte balance. However, its physiological functions reach far beyond that, and include the synthesis of relevant enzymatic and hormonal factors and regulation of the degradation and excretion of metabolic end-products as well. CKD is a state that can involve either an excess or deficiency of certain substances or physiological processes in the body, resulting in an imbalance that profoundly disturbs homeostasis. In CKD, one side of the coin is a state of noxious excess, a “too much” of something. This is especially notable with respect to substances with decreased clearance, and thus renal retention, e.g., uremic toxins, water, electrolytes, creatinine levels and others. Higher-than-normal concentrations of certain substances might fuel a cascade of adaptive systemic processes in certain metabolic or endocrine pathways in order to maintain homeostasis as long as possible despite declining renal function. Phosphate retention and the consecutive disorders in the FGF23-PTH-bone axis serve as prominent examples for such a link between renal retention and the consequent profound systemic multi-organ consequences [4]. Further, CKD represents a state of chronic overactive sympathetic nervous system, which has many negative effects on the body, including arterial hypertension, insulin resistance and further damage to the kidneys [5]. On the other hand, CKD is characterized by various states of deficiency and insufficiency as well as critical shortages. There are at least four basic mechanisms responsible for inducing shortages or an undersupply caused by CKD: (I) Excess renal loss of plasma components such as proteins by glomerular filtration barrier leakage, which can eventually lead to hypoalbuminemia, loss of antithrombotic proteins and hypogammaglobulinemia (the so-called nephrotic syndrome) [6]. The Fanconi-syndrome is another example of excess renal loss leading to systemic deficiencies. (II) Reduced hormonal activities: In CKD, the kidneys gradually lose their ability to produce erythropoietin, leading to a decrease in red blood cell production and, consequently, anemia [7]. (III) Malnutrition, dietary deficiencies and/or gastrointestinal malabsorption are also relevant comorbidities of CKD [8]. (IV) The increased expression of certain factors and hormones in CKD, which in turn can suppress other physiological processes. As an example, FGF23 serum levels become elevated in CKD to increase the excretion of phosphate, but also reduce the activity of renal 1,25-dihydroxylase (CYP27B1), which can lead to a deficiency in active vitamin D and hypocalcemia [9]. Many CKD-related diseases, such as chronic kidney disease–mineral and bone disorder (CKD-MBD) or renal anemia, share several or all four of the above-mentioned pathophysiological mechanisms. This underlines the complexity of renal pathophysiology and CKD-related disorders. In the care of patients with CKD, efforts to therapeutically replenish deficiencies are routine in daily clinical practice. Notably, it is often insufficient to simply aim at the restoration of physiological levels or concentrations when referring to the treatment of CKD-associated deficiencies. Again, CKD-MBD and renal anemia are instructive examples. Native vitamin D deficiency is highly prevalent in CKD and is among the driving forces of hyperparathyroidism [10]. Trial data have clearly shown that an augmentation of vitamin D levels higher than generally accepted as “sufficient” is necessary (i.e., >50 ng/mL) in order to lower PTH levels in renal hyperparathyroidism [11]. In contrast, 25-hydroxyvitamin D levels as low as 20 ng/mL presumably meet the requirements in the vast majority of the general population [12]. A similar discrepancy is present in renal anemia: The iron metabolism target levels for transferrin saturation and ferritin to which iron replenishment should lead in CKD patients (in other words the level of sufficiency in CKD patients) are generally considered to be much higher [13] than the cut-off levels, below which a state of iron deficiency is defined in the general population [14,15]. Another example is the vitamin k deficiency in CKD, which is common due to food restrictions, gut dysbiosis, and drugs. Moreover, experimental work shows that CKD is associated with disturbances in transportation, tissue storage and enzymatic activation of vitamin K [16]. Vitamin K deficiency has been associated with accelerated vascular calcification. Recently, growing evidence has suggested that supraphysiological administration of vitamin K1 could be a safe tool to prevent progression of calcifications [16,17,18]. Hence, the guideline recommendations regarding nutritional intake of certain substances or therapeutic replenishment strategies developed for the general population must be carefully evaluated with respect to CKD patients. What might be appropriate in the general population to avoid dietary or therapeutic undersupply and to refill body storages to overcome deficiencies might be insufficient in the setting of CKD. In summary, CKD requires a novel definition of optimal balance between “too much” and “too little”.
